# Supplementary material for: Topographic axonal projection at single-cell precision supports local retinotopy in the mouse superior colliculus
Source: Nat Commun. 2023 Nov 16;14:7418. doi: 10.1038/s41467-023-43218-x (PMC10654506; doi:10.1038/s41467-023-43218-x)
Supplement: Supplementary file 1 — Supplementary Information [file 41467_2023_43218_MOESM1_ESM.pdf]

Topographic axonal projection at single-cell precision supports local retinotopy in the mouse superior colliculus

Dmitry Molotkov[1], Leiron Ferrarese[1], Tom Boissonnet[1,2,3], Hiroki Asari[1*]

1. Epigenetics and Neurobiology Unit, EMBL Rome, European Molecular Biology Laboratory, Monterotondo, 00015, Italy
2. Collaboration for joint PhD degree between EMBL and Université Grenoble Alpes, Grenoble Institut des Neurosciences, La Tronche, 38700, France
3. Current address: Center for Advanced Imaging, Heinrich-Heine-Universität Düsseldorf, Düsseldorf, 40225, Germany

* Corresponding author: asari@embl.it
These authors contributed equally: Dmitry Molotkov, Leiron Ferrarese.

Supplementary Information

- Supplementary Figure 1
- Supplementary Figure 2
- Supplementary Figure 3
- Supplementary Movie 1

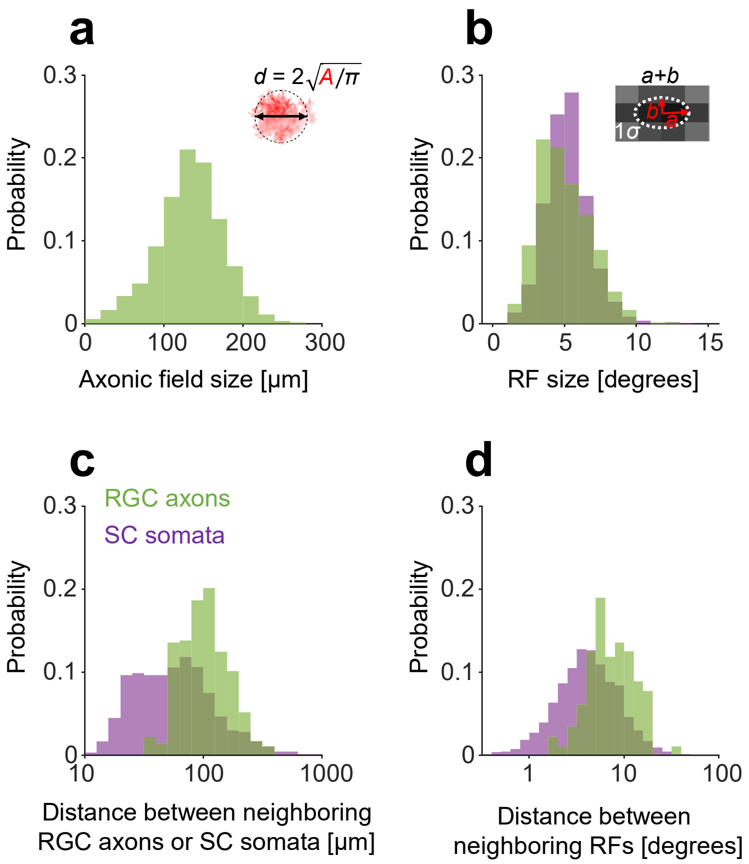

Supplementary Figure 1: Probability distributions of axonic and receptive field statistics. a Size of the identified retinal ganglion cell (RGC) axon terminals, $d = 2(A/\pi)^{0.5}$, where A is the axonic field area from the constrained non-negative matrix factorization analysis (135 ± 25 µm, median ± median absolute deviation; n = 969). **b** Receptive field (RF) size of RGC axons (green; 4.8 ± 1.2 degrees; n = 719) and superior colliculus (SC) somata (purple; 5.1 ± 0.9 degrees; n = 1191), measured as the mean of long- and short-diameters (equivalently, the sum of long- and short-radii, a and b, respectively) of the two-dimensional Gaussian profile at 1 σ fitted to the RF (e.g., Fig. 2E). **c** Distance between neighboring RGC axon centers (green; 100 ± 30 µm; n = 761 pairs) or SC somata (purple; 56 ± 28 µm; n = 2292 pairs). Neighboring pairs are identified by the Delaunay triangulation analysis (e.g., Fig. 3c and Supplementary Fig. 3f). **d** Distance between neighboring RF centers of RGC axons (green; 7.2 ± 2.7 degrees; n = 776 pairs) or SC somata (purple; 4.1 ± 1.9 degrees; n = 2287 pairs). Neighboring pairs are identified by the Delaunay triangulation analysis (e.g., Fig. 3d and Supplementary Fig. 3g). Source data are provided as a Source Data file.

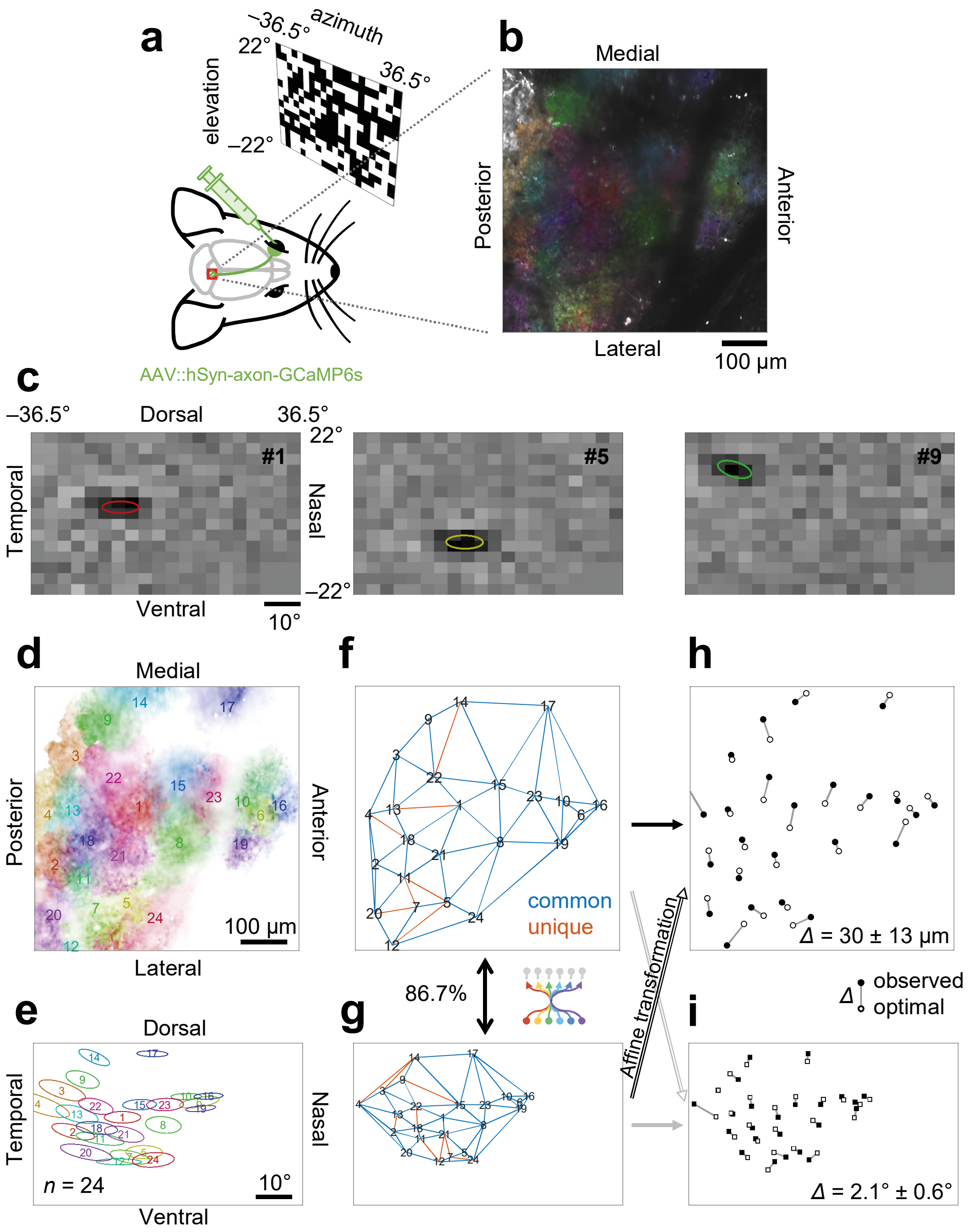

Supplementary Figure 2: Another example of precise tiling of retinal ganglion cell axons in the mouse superior colliculus. Figure panels are shown in the same format as in Figs. 2 and 3. **a** Schematic diagram of retinal ganglion cell (RGC) axonal imaging in the superior colliculus (SC). **b** Average intensity projection of representative imaging data, overlaid with detected RGC axonal patches (n = 24; color-coded). **c** Receptive fields (RFs) of three representative RGC axonal patches. Ellipse, 1 standard deviation (SD) Gaussian profile. **d,e** Tiling patterns of RGC axons (**d**) and their corresponding RFs (**e**; 1 SD Gaussian profiles with the same color-code as in **b**). **f,g** Delaunay triangulation of the RGC axon centers (**f**; from **d**) and the RF centers (**g**; from **e**), showing a good agreement between them (86.7%; blue, common edges in both patterns; red, unique edges only in either pattern). **h,i** Comparison between the observed and retinotopically ideal tiling patterns of RGC axons (**h**; Δ = 30 ± 13 µm) or their RFs (**i**; Δ = 2.1 ± 0.6 degrees).

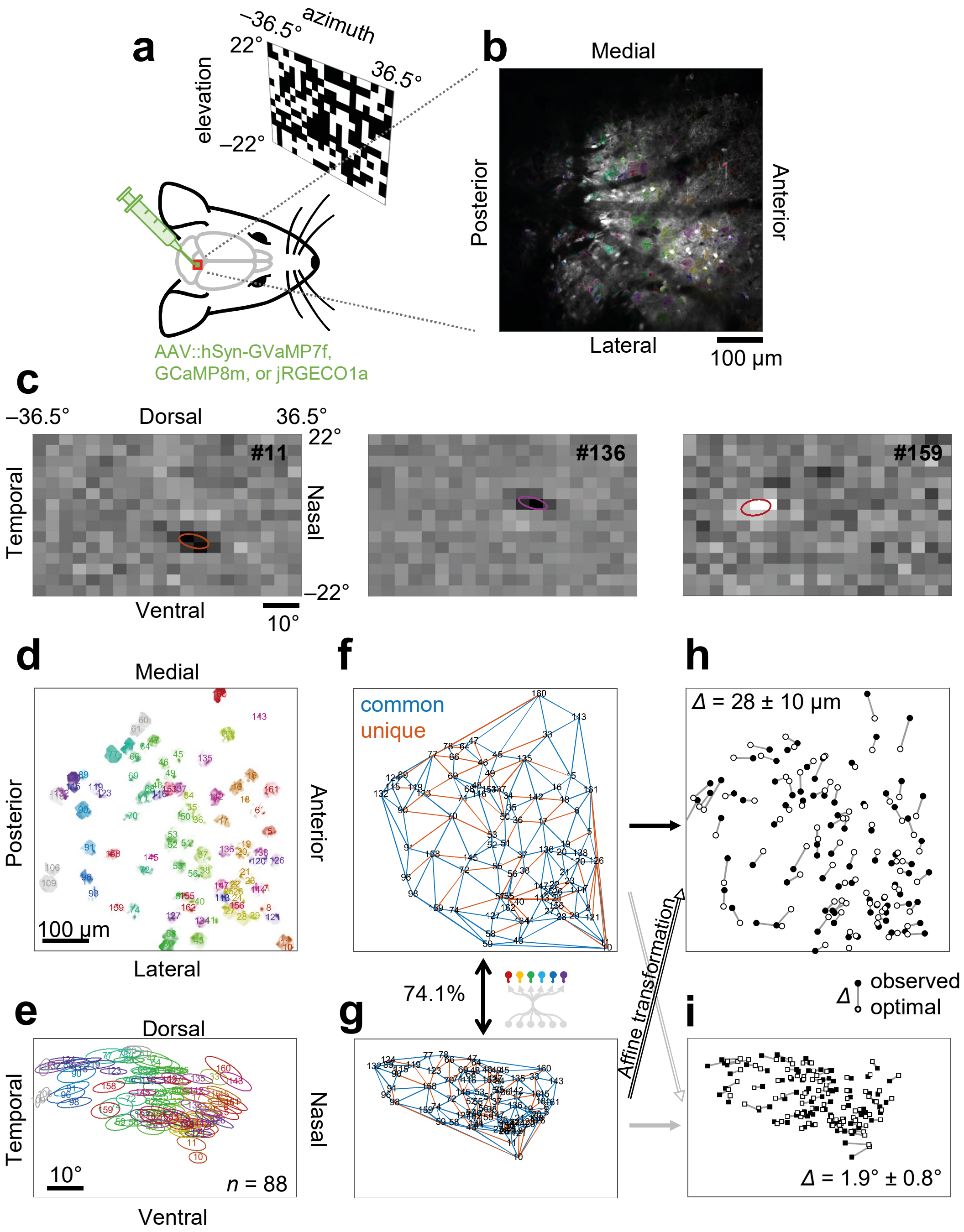

Supplementary Figure 3: Tiling pattern analysis of local neurons in the mouse superior colliculus. Figure panels are shown in the same format as in Figs. 2 and 3. **a** Schematic diagram of the experimental set up for superior colliculus (SC) somatic imaging. **b** Average intensity projection of representative imaging data, overlaid with detected SC somata (n = 88; color-coded). **c** Representative receptive fields (RFs) of local SC neurons (from left to right: #11, #136, #159) from reverse-correlation analysis. Ellipse, 1 standard deviation (SD) Gaussian profile. **d,e** Tiling pattern of SC somata (**d**) from a representative recording session (**b**) and their corresponding RF tiling pattern (**e**; 1 SD Gaussian profiles with the same color-code as in **b**). **f,g**: Delaunay triangulation of the SC somatic locations (**f**; from **d**) and the RF centers (**g**; from **e**), showing a good agreement between them (74.1%; blue, common edges in both patterns; red, unique edges only in either pattern). **h,i**: Comparison between the observed and retinotopically ideal tiling patterns of SC somata (**h**; Δ = 28 ± 10 μm) or their RFs (**i**; Δ = 1.9 ± 0.8 degrees).

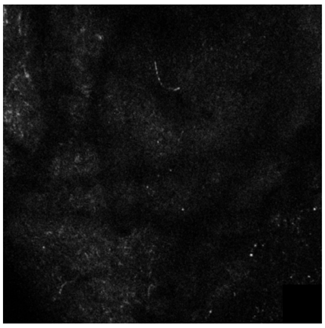

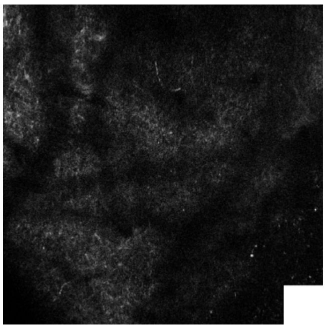

Supplementary Movie 1: *In vivo* two-photon calcium imaging of retinal ganglion cell axon terminals in the mouse superior colliculus. A snippet of rigid motion-corrected movie showing the activity of retinal ganglion cell (RGC) axons in the mouse superior colliculus (SC) in response to the random checkerboard stimuli (sampling rate, 15.4 Hz; averaged every 5 frames for noise reduction, hence sped up by 5 times). Remaining non-rigid motion was further corrected before signal extraction by CaImAn[24] (see Methods for details). A black square overlaid at the bottom-right corner (example frame on the left) turns white when the visual stimulus is on (example frame on the right). See Fig. 2 for the segmentation and Fig. 3 for the tiling pattern analysis.
