# Supplementary material for: Topographic axonal projection at single-cell precision supports local retinotopy in the mouse superior colliculus
Source: Nat Commun. 2023 Nov 16;14:7418. doi: 10.1038/s41467-023-43218-x (PMC10654506; doi:10.1038/s41467-023-43218-x)
Supplement: Supplementary file 4 — Reporting Summary [file 41467_2023_43218_MOESM4_ESM.pdf]

nature portfolio

Corresponding author(s): Hiroki Asari

Last updated by author(s): Oct 26, 2023

Reporting Summary

Nature Portfolio wishes to improve the reproducibility of the work that we publish. This form provides structure for consistency and transparency in reporting. For further information on Nature Portfolio policies, see our Editorial Policies and the Editorial Policy Checklist.

Statistics

For all statistical analyses, confirm that the following items are present in the figure legend, table legend, main text, or Methods section.

n/a	Confirmed	
☐	☒	The exact sample size (*n*) for each experimental group/condition, given as a discrete number and unit of measurement
☐	☒	A statement on whether measurements were taken from distinct samples or whether the same sample was measured repeatedly
☐	☒	The statistical test(s) used AND whether they are one- or two-sided<br>*Only common tests should be described solely by name; describe more complex techniques in the Methods section.*
☒	☐	A description of all covariates tested
☒	☐	A description of any assumptions or corrections, such as tests of normality and adjustment for multiple comparisons
☐	☒	A full description of the statistical parameters including central tendency (e.g. means) or other basic estimates (e.g. regression coefficient) AND variation (e.g. standard deviation) or associated estimates of uncertainty (e.g. confidence intervals)
☐	☒	For null hypothesis testing, the test statistic (e.g. *F*, *t*, *r*) with confidence intervals, effect sizes, degrees of freedom and *P* value noted<br>*Give P values as exact values whenever suitable.*
☒	☐	For Bayesian analysis, information on the choice of priors and Markov chain Monte Carlo settings
☒	☐	For hierarchical and complex designs, identification of the appropriate level for tests and full reporting of outcomes
☒	☐	Estimates of effect sizes (e.g. Cohen's *d*, Pearson's *r*), indicating how they were calculated

Our web collection on statistics for biologists contains articles on many of the points above.

Software and code

Policy information about availability of computer code

Data collection	Data were recorded with Scientifica SciScan 1.3 image acquisition software. Visual stimuli were presented with QDSpy 0.77beta software (Franke et al., 2019).
Data analysis	The data analyses were done in Python 3 and MATLAB R2018b-R2023a (Mathworks) using custom codes; FIJI 1.51-1.54 (NIH) and CaImAn 1.0.1 (Giovannucci et al., 2019) for calcium image data preprocessing. All custom codes are available on FIGSHARE along with the relevant data: https://doi.org/10.6084/m9.figshare.24158658

For manuscripts utilizing custom algorithms or software that are central to the research but not yet described in published literature, software must be made available to editors and reviewers. We strongly encourage code deposition in a community repository (e.g. GitHub). See the Nature Portfolio guidelines for submitting code & software for further information.

Data

Policy information about availability of data

All manuscripts must include a data availability statement. This statement should provide the following information, where applicable:

- Accession codes, unique identifiers, or web links for publicly available datasets
- A description of any restrictions on data availability
- For clinical datasets or third party data, please ensure that the statement adheres to our policy

The data the support the findings of this study are available on FIGSHARE: https://doi.org/10.6084/m9.figshare.24158658
Source data are provided with this paper.

Research involving human participants, their data, or biological material

Policy information about studies with human participants or human data. See also policy information about sex, gender (identity/presentation), and sexual orientation and race, ethnicity and racism.

Reporting on sex and gender	N/A
Reporting on race, ethnicity, or other socially relevant groupings	N/A
Population characteristics	N/A
Recruitment	N/A
Ethics oversight	N/A

Note that full information on the approval of the study protocol must also be provided in the manuscript.

Field-specific reporting

Please select the one below that is the best fit for your research. If you are not sure, read the appropriate sections before making your selection.

☒ Life sciences ☐ Behavioural & social sciences ☐ Ecological, evolutionary & environmental sciences

For a reference copy of the document with all sections, see nature.com/documents/nr-reporting-summary-flat.pdf

Life sciences study design

All studies must disclose on these points even when the disclosure is negative.

Sample size	No statistical method was used to predetermine the sample size. The sample was considered adequate and consistent with the literature in the field (e.g., PMID: 16033902, 30174186).
Data exclusions	No primary data were excluded from this study.
Replication	Experimental results were reproducible across mice (up to 5 recording sessions/animal).
Randomization	Randomized stimuli were presented during recordings. Randomized method was used for calculating the chance level tiling pattern match (Figure 4) and for the modeling analyses (Figures 5 and 6). Mice were not randomized as they all stem from controlled C57BL/6J background, but picked randomly from the colony without any biased selection criteria.
Blinding	Blinding was not applied because a detailed (non-blinded) examination of all probed parameters for each recording was required.

Reporting for specific materials, systems and methods

We require information from authors about some types of materials, experimental systems and methods used in many studies. Here, indicate whether each material, system or method listed is relevant to your study. If you are not sure if a list item applies to your research, read the appropriate section before selecting a response.

Materials & experimental systems

n/a	Involved in the study
☒	☐ Antibodies
☒	☐ Eukaryotic cell lines
☒	☐ Palaeontology and archaeology
☐	☒ Animals and other organisms
☒	☐ Clinical data
☒	☐ Dual use research of concern
☒	☐ Plants

Methods

n/a	Involved in the study
☒	☐ ChIP-seq
☒	☐ Flow cytometry
☒	☐ MRI-based neuroimaging

Animals and other research organisms

Policy information about studies involving animals; ARRIVE guidelines recommended for reporting animal research, and Sex and Gender in Research

Laboratory animals	C57BL/6J mice were used at around 6-10 weeks of age at the time of the first surgery, and between 12-24 weeks of age at the time of imaging experiments. Mice were kept on a 12-h light / 12-h dark cycle (ambient temperature, around 21 degrees Celsius; humidity, 45-60%), and given water and food ad libitum.
Wild animals	No wild animals were used in this study.
Reporting on sex	35 female mice were used in this study. No male mice were used in this study due to difficulties to keep them in an head-fixed condition long enough for awake recordings. We nevertheless expect that the results do not depend on the sex of the animals.
Field-collected samples	No field collected samples were used in the study.
Ethics oversight	All experiments involving animals were performed under the license 233/2017-PR from the Italian Ministry of Health, following protocols approved by the Institutional Animal Care and Use Committee at European Molecular Biology Laboratory.

Note that full information on the approval of the study protocol must also be provided in the manuscript.
